# Tropical Diseases in the United States: Beyond Poverty – Advancing an Ecological Framework in Tropical Medicine

**DOI:** 10.4269/ajtmh.24-0251

**Published:** 2024-07-23

**Authors:** Michael E. DeWitt, John W. Sanders

**Affiliations:** ^1^ Section on Infection Diseases, Department of Medicine, Wake Forest University School of Medicine, Winston-Salem, North Carolina;; ^2^ Center for the Study of Microbial Ecology and Emerging Diseases, Wake Forest University School of Medicine, Winston-Salem, North Carolina;; ^3^ Department of Biology, Wake Forest University, Winston-Salem, North Carolina

In the realm of infectious diseases, understanding the complex interplay between humans, society, and their environment is essential. This is especially true for the infectious diseases we historically classify as “tropical diseases.” A study by Blackburn et al., published in this issue of the American Journal of Tropical Medicine and Hygiene, highlights the persistence of such infections in the United States and provides a compelling illustration of the social and environmental dynamics that facilitate that persistence.^1^ The research team investigated the association between environmental presence of parasites and social vulnerability in the Southern United States. By employing advanced molecular detection techniques, they meticulously analyzed soil samples from five low-income communities across different states. Their findings reveal a striking correlation: regions with the highest rates of social vulnerability and poverty also bear the heaviest burden of parasitic contamination. This study sheds light on the prevalence of parasites in these communities and underscores the profound influence of socio-economic factors on the dynamics of infectious disease transmission.

The findings of Blackburn et al., also highlight how disease can be intrinsically linked to environmental conditions. In their study they found a high prevalence in soil of multiple pathogens, including *Blastocystis spp* (19.0%), *Toxocara cati* (6.0%), *Toxocara canis* (3.6%), *Strongyloides stercoralis* (2.0%), *Trichuris trichiura* (1.8%), *Ancylostoma duodenale* (1.4%), *Giardia intestinalis* (1.4%), *Cryptosporidium spp* (1.0%), *Entamoeba histolytica* (0.20%), and *Necator americanus* (0.20%).^1^ The authors describe parasite prevalence as ‘environmental contamination,’ which appears to correlate with areas with a high social vulnerability index (SVI). The SVI incorporates multiple factors including poverty rate, access to transportation, and housing density, and gauges community resilience to external stresses such as pandemics or natural disasters.^2^ Many of the parasites identified are associated with primary animal hosts or reservoir species, both domestic and wild. It is not surprising that residents in areas with high SVI scores might encounter animals and their waste more frequently than those living in other areas. There are clearly correlations between SVI and the environmental justice index, another index designed to measure cumulative health impacts related to environmental burdens.^3^^,^^4^

The factors inherently highlighted by this study, such as poverty, environmental conditions, and human and animal behaviors are crucial for understanding disease risk. Scheiner defined the ‘domain of ecology’ as the “study of the spatial and temporal patterns in the distribution and abundance of organisms, including their causes and consequences.”^5^ The assessment of the persistence of ‘tropical infections’ in the United States aptly fits in that domain.

Ecology is the study of the interactions between organisms and their environments. It encompasses the distribution and abundance of organisms and the dynamics of populations, communities, and ecosystems. Ecologists investigate how changing dynamics in resources influence the diversity and stability of biological communities. Ecology encompasses the effects of human activity on the environment, climate change, and habitat change and how these may shape the connections and interactions between species. This discipline is crucial for understanding the natural world and for informing policy decisions that help manage and protect the environment. Going beyond the concept of One Health, ecology examines the dynamics and coexistence amongst all species, along with their parasites. An ecologic framework unites the multidisciplinary approach intrinsic to the study of tropical medicine that is so clearly reflected in the membership of ASTMH.

Tropical medicine has been at the forefront of the recognition of the interplay between ecology and infectious diseases. Since Ronald Ross’ recognition of the role of the population sizes of mosquitos required to sustain malaria transmission, the roots of the field have been fundamentally framed by ecological thinking.^6^ Furthermore, many of the models used by the infectious disease community to shape policy emerged from ecological thinking, including Kermack and McKendrick’s 1927 landmark publication on compartmental models to Anderson and May’s 1991 tome, the *Infectious Diseases of Humans.*^7^^,^^8^ A fundamental understanding of the ecology of the *Anopheles* mosquito contributed importantly to the elimination of endemic malaria in the United States.^9^ Ecological insights applied to infectious diseases continue to inform our understanding of the spread of Lyme disease and how land use and climate influence the abundance and distribution of pathogens and their vectors.^10^^–^^15^ These ecological insights extend to understanding the characteristics of emerging pathogens acquired from wildlife and pets, and to informing the design of interventions, including mass drug administration and chemoprophylaxis.^16^^–^^19^

With global climate change, international trade, and land use changes, a return to the ecological underpinnings of the study of infectious diseases has never been more important. As the pattern and frequency of our environmental contacts change, we see the return of previously endemic pathogens. This has been evident in the re-establishment of mosquito-borne diseases in the United States. The resurgence and spread of *Aedes*-borne diseases in the Americas, including dengue, Zika, and chikungunya, have been significantly influenced by climatic factors that affect both the pathogens and their mosquito vectors. Dengue has a long-standing history in the United States, with outbreaks recorded as early as the colonial era, especially in southern states. Efforts to eradicate the primary mosquito vector, *Aedes aegypti*, drastically reduced dengue cases by the 1970s. Yet, the disease re-emerged in the 1980s, fueled by decreased mosquito control, urbanization, and global travel and trade, leading to sporadic outbreaks in Hawaii, Texas, and Florida. Local, autochthonous transmission of chikungunya in 2013 and Zika in 2017 has also been documented.^20^ Similarly, malaria has reappeared, with autochthonous cases of *Plasmodium vivax* infection reported in Texas and Florida, highlighting ongoing challenges as the *Anopheles* vector persists in the southeast United States. Climate change projections suggest potential expansion of this vector’s range, increasing the risk of malaria becoming endemic again in the southern U.S.^17^^,^^21^

In addition to the return of previously endemic pathogens, we have seen an increase in the range of preexisting pathogens. This includes a rapid expansion to the area in which leprosy has been diagnosed.^22^ This expansion may be due to the expanding range of armadillos, a known reservoir for *Mycobacterium leprae,* likely driven by human-induced environmental changes.^23^^,^^24^ Similarly, recent studies have shown increasing range of soil-borne dimorphic fungi, potentially due to human induced land use change, with high nitrogen content soils associated with the presence of *Histoplasma*.^25^^,^^26^

There is an increasing recognition of pathogens historically considered “tropical,” in part due to heightened recognition with improved diagnostics. *Orientia spp*, traditionally confined to the ‘tsutsugamushi triangle’ were found in chiggers across multiple locations in North Carolina in 2023, suggesting that the organisms have been introduced to the United States.^27^ Three cases of melioidosis caused by presumed local environmental exposure to *Burkholderia pseudomallei* in Mississippi suggest new recognition or possibly new introduction into this region. The importation of animals and the presence of domestic sandflies may allow for *Leishmania mexicana* to establish among the local dog population in Texas and beyond.^25^^,^^28^^–^^31^ Similarly, a recent systematic review found evidence for 76 confirmed or suspected autochthonous of human Chagas diseases. The United States is home to at least 11 species of triatomines, eight of which are capable of harboring *Trypanosoma cruzi,* and recent studies have shown that dogs may again play an important role in sylvatic transmission and maintenance of the *T. cruzi* population.^32^^,^^33^

Infectious disease ecology offers the tools of population dynamics, community ecology, and invasion biology to inform our interventions against new pathogens.^34^^,^^35^ This includes understanding the risk of dispersal of the pathogen into a new host population within a new niche. This approach includes examining where new introductions of pathogens are likely to occur, be they through travel and trade, expansion in range, new availability of vectors, or changing contact patterns driven by socio-demographics and climate change, allowing us to estimate the probability of establishment and persistence within that local context. Finally, we can examine the risk of regional spread and put into place mitigations tailored to the local ecological dynamics to contain further spread. Time may tell whether organisms we have described as reemerging, expanding in range, and newly emerging have become endemic.

Blackburn et al.,^1^ build on the literature indicating that those in poverty are more likely to be exposed and infected with infectious diseases.^36^^–^^38^ As Rudolf Virchow noted in his landmark study of a typhus epidemic in 1848, social and economic conditions have a large impact on the health of humans and the spread of infectious diseases.^39^ Furthermore, those in poverty are more likely to bear the brunt of climate change.^40^ Poor Americans will be disproportionately impacted by rising sea levels which bring damper soil, more conducive to hosting parasites and other pathogens, increasing potential contact with humans.^41^^,^^42^

The dynamics of tropical infectious diseases are just that—dynamic. One weakness of the study by Blackburn et al.,^1^ is that their samples were limited to a single cross section across multiple counties.^1^ Greater longitudinal sampling would allow for us to understand how the prevalence and abundance of the pathogens which they detected changed through time. Similarly, Blackburn et al.^1^ cannot determine if higher prevalence of parasites in the soil result in higher disease morbidity. While the authors analyzed nearly 500 samples across five communities, broader geographic sampling would be required to gain a more holistic view of the dynamics of pathogen exposure. A broader sampling of communities would also be required to better infer the role of poverty on exposure to pathogens, given that only poor communities were included in this study. Socio-demographics and poverty are complicated concepts, with heterogeneity even at the community level, suggesting caution with conclusions based on broad population rates. Despite these limitations, the authors have provided an exceptional framework for future sampling studies.

Given the dynamic, interconnected nature of our world, it is essential to integrate ecological insights across all disciplines involved in infectious diseases. Researchers and practitioners in tropical medicine must continue to lead in examining disease emergence, re-emergence, distribution, and ultimate persistence through an ecological lens.
